# Supplementary material for: Causal Relationship between Aging and Anorexia Nervosa: A White-Matter-Microstructure-Mediated Mendelian Randomization Analysis
Source: Biomedicines. 2024 Aug 16;12(8):1874. doi: 10.3390/biomedicines12081874 (PMC11351342; doi:10.3390/biomedicines12081874)
Supplement: Supplementary file 1 [file biomedicines-12-01874-s001.zip › Supplementary Figure/Figure S4/Singe SNP plot of MR analysis for TL to ubm-b-1914.pdf]

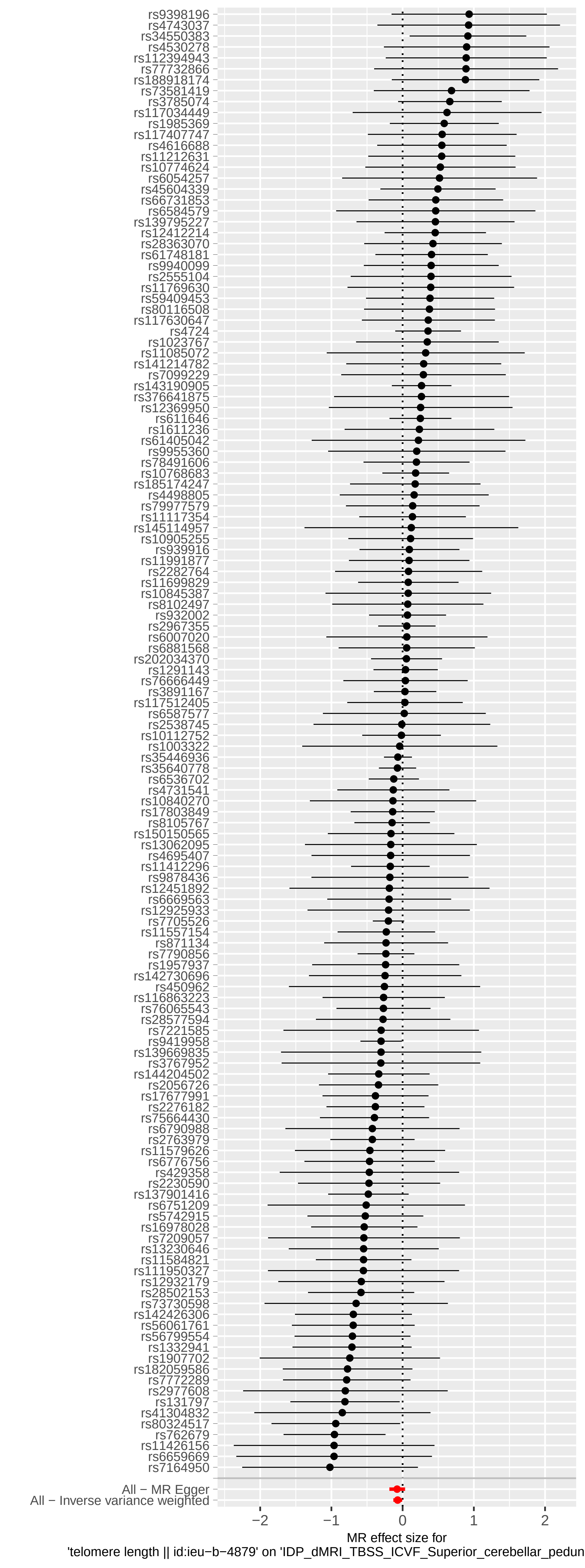
rs9398196
rs4743037
rs34550383
rs4530278
rs112394943
rs77732866
rs188918174
rs73581419
rs3785074
rs117034449
rs1985369
rs117407747
rs4616688
rs11212631
rs10774624
rs6054257
rs45604339
rs66731853
rs6584579
rs139795227
rs12412214
rs28363070
rs61748181
rs9940099
rs2555104
rs11769630
rs59409453
rs80116508
rs117630647
rs4724
rs1023767
rs11085072
rs141214782
rs7099229
rs143190905
rs376641875
rs12369950
rs611646
rs1611236
rs61405042
rs9955360
rs78491606
rs10768683
rs185174247
rs4498805
rs79977579
rs11117354
rs145114957
rs10905255
rs939916
rs11991877
rs2282764
rs11699829
rs10845387
rs8102497
rs932002
rs2967355
rs6007020
rs6881568
rs202034370
rs1291143
rs76666449
rs3891167
rs117512405
rs6587577
rs2538745
rs10112752
rs1003322
rs35446936
rs35640778
rs6536702
rs4731541
rs10840270
rs17803849
rs8105767
rs150150565
rs13062095
rs4695407
rs11412296
rs9878436
rs12451892
rs6669563
rs12925933
rs7705526
rs11557154
rs871134
rs7790856
rs1957937
rs142730696
rs450962
rs116863223
rs76065543
rs28577594
rs7221585
rs9419958
rs139669835
rs3767952
rs144204502
rs2056726
rs17677991
rs2276182
rs75664430
rs6790988
rs2763979
rs11579626
rs6776756
rs429358
rs2230590
rs137901416
rs6751209
rs5742915
rs16978028
rs7209057
rs13230646
rs11584821
rs111950327
rs12932179
rs28502153
rs73730598
rs142426306
rs56061761
rs56799554
rs1332941
rs1907702
rs182059586
rs7772289
rs2977608
rs131797
rs41304832
rs80324517
rs762679
rs11426156
rs6659669
rs7164950
All − MR Egger
All − Inverse variance weighted
−2
−1
0
1
2
MR effect size for
'telomere length || id:ieu−b−4879' on 'IDP_dMRI_TBSS_ICVF_Superior_cerebellar_pedun
